# Characterization of Intestinal Microbiota and Probiotics Treatment in Children With Autism Spectrum Disorders in China

**DOI:** 10.3389/fneur.2019.01084

**Published:** 2019-11-05

**Authors:** Manman Niu, Qinrui Li, Jishui Zhang, Fang Wen, Weili Dang, Guiqin Duan, Haifeng Li, Wencong Ruan, Pingri Yang, Chunrong Guan, Huiling Tian, Xiaoqing Gao, Shaobin Zhang, Fangfang Yuan, Ying Han

**Affiliations:** ^1^Department of Pediatrics, Peking University First Hospital, Beijing, China; ^2^Department of Pediatrics, Beijing Tiantan Hospital, Capital Medical University, Beijing, China; ^3^Department of Pediatrics, Peking University People's Hospital, Beijing, China; ^4^National Center for Children‘s Health, Beijing Children's Hospital, Capital Medical University, Beijing, China; ^5^Children's Encephalopathy Diagnosis and Rehabilitation Center, The First Affiliated Hospital of Henan University of Chinese Medicine, Zhengzhou, China; ^6^Center of Children Psychology and Behavior, Henan Maternal and Child Health Hospital, Zhengzhou, China; ^7^The Children's Hospital Zhejiang University School of Medicine, Hangzhou, China; ^8^Department of Pediatric Rehabilitation, Jiningshi Renchengqu Women's and Children's Health Care Hospital, Jining, China; ^9^Department of Pediatric Rehabilitation, Linyishi Women's and Children's Hospital, Linyi, China; ^10^Beijing Gutgene Technology Co. Ltd, Beijing, China; ^11^Xinxiang Central Hospital, Xinxiang, China

**Keywords:** autism spectrum disorder, intestinal microbiota, probiotics treatment, children, China

## Abstract

**Background:** Most previous studies have found that human intestinal microbiota affect the symptoms of autism spectrum disorder (ASD), especially gastrointestinal (GI) symptoms, but regarding this, there is limited data of non-western ethnicity. Probiotics can reconstitute the host intestinal microbiota and strengthen gastrointestinal function, however, clinical data proving the effect of probiotics treatment on ASD is lacking.

**Methods:** This study explored the significant differences between ASD and neurotypical (NT), and the improvement of applied behavior analysis (ABA) training in combination with probiotics, vs. ABA training only.

**Results:** We found significant differences between the ASD group and the NT group in the evenness of the intestinal microbiota and the relative abundance of the bacterial phyla and genus. At the phylum level, relative abundance of *Bacteroidetes* in the ASD group was significantly lower than in the NT group. At the genus level, the relative abundance of *Bacteroides, Bifidobacterium, Ruminococcus, Roseburia*, and *Blautia* in the ASD group was significantly lower than that in the NT group. After a 4-week ABA training program in combination with probiotics treatment, the *ATEC* and GI scores decreased more than the control group with ABA training only.

**Conclusion:** Our findings suggest that intestinal microbiota is different between the NT children and the ASD children with or without GI problems. In combination with ABA training, probiotics treatment can bring more benefit to ASD children. Clinical trials with a more rigorous design and larger sample size are indispensable for further validation.

## Introduction

Autism spectrum disorder (ASD) refers to a group of neurodevelopmental disorders with multiple, heterogenous causes. ASD is characterized by stereotyped behavior, language, and social interaction disorders. The incidence of ASD in children from 1.6 to 8 years old in China is approximately 39.23/10,000 (1). The etiology of ASD is complex; both heredity and the environmental are involved (2). Studies have found that human intestinal microbiota affect the human brain and may affect human behavior and mental health through the gut-brain axis (3–7). The most common non-neurological indications in children with ASD are gastrointestinal symptoms (GI) such as constipation and diarrhea (8). Many studies have shown that the intestinal microbiota of autistic children with GI is different from that of neurotypical (NT) children (9–12), and the GI in the children with ASD corrects with their autistic manifestations (13).

Currently there is no cure for ASD. Children with ASD require long-term applied behavior analysis (ABA) training to improve their symptoms. Probiotics treatment can reconstitute the host intestinal microbiota, restore microbiota homeostasis, and strengthen gastrointestinal function. Animal models (14) and preliminary clinical trials have shown that probiotics treatment can alleviate ASD symptoms (15–17). Probiotics treatment may be a suitable therapy for ASD (18–20). Most previous studies of the relationship between ASD and GI have been conducted on participants of Western countries; research on children with ASD in China is scarce (21, 22). Therefore, this study aims to study the intestinal microbiota of children with ASD in China, and both ASD children with and without GI are included. Children with ASD are treated with probiotics and the clinical outcomes are investigated.

## Materials and Methods

### Subject Recruitment and Sample Collection

The ethics committee of Peking University has reviewed and approved this study. It has been registered in the Chinese Clinical Trial Registry (ChiCTR1900023609) and all patients have signed the written informed consent. A total of 114 children with ASD (diagnosed according to Diagnostic and Statistical Manual of Mental Disorders, 5th Edition) (23) were recruited from hospital pediatric departments, hospital neurodevelopmental departments, and autistic rehabilitation institutions in Beijing, Shandong, Henan, and Zhejiang. In the meantime, a total of 40 neurotypical (NT) children from regular preschools were selected as the negative control group. ASD enrollment criteria were as follows: (1) diagnosis of ASD according to DSM-V; (2) age 3–8 years old; (3) no kinship between participants; (4) no antibiotics, probiotics, or other gastrointestinal treatments within 1 month prior to enrollment. ASD exclusion criteria were as follows: (1) No diagnosis of ADHD or other non-ASD neurological peculiarity, which cause autism-like manifestations; (2) children with other mental illnesses, brain organic diseases, severe liver and kidney diseases, and cardiovascular diseases; (3) children's guardians did not give permission for participation or did not complete follow-up. NT children were selected using the following criteria: (1) age 3–8 years old, normal development; (2) no family history of ASD; (3) children's guardians agreed to participate in the study and signed informed consent; (4) no antibiotics, or other gastrointestinal treatments within 1 month before enrollment.

First defecations of the day were collected at home by their parents. The DNA samples were extracted for PCR using OMEGA E.Z.N.A Stool DNA Kit and were quantified by Nanodrop. PCR was carried out on GeneAmp® 9700 with Phusion High-Fidelity PCR Master Mix, the primers were 341F and 806R.The mixture of purified PCR products was generated for Next-generation sequencing (NGS) library using Agencourt AMPure XP 60 ml Kit following manufacturer's recommendations. The library quality was quantified by Qubit dsDNA HS Assay Kitwith the Qubit 2.0 fluorometer system. The multiplexed amplicons were sequenced using the Illumina MiSeq platform to generate 300 bp paired-end reads. *De novo* operational taxonomic unit picking was done using QIIME2 software (24).

### Probiotics Treatment

Thirty-seven children with ASD were treated with 4-week applied behavior analysis (ABA) training in combination with probiotics. Probiotics are lyophilized, water-soluble powder that contains 6 strains of bacteria; each strain has 1 billion CFU/gram. The dosage is 6 g per day (36 billion CFU in total). Twenty-eight other children with ASD were randomly selected as a control group and treated with ABA training alone. During the study, both groups were prohibited from the use of antibiotics, other probiotics, prebiotics, or any other treatment that might alter the intestinal microbiota.

### Evaluation of ASD-Related and Gastrointestinal Symptoms

ASD-related symptoms were assessed using the Autism Treatment Evaluation Checklist (*ATEC*) before and after probiotics treatment. Clinical assessment information for children with ASD was provided by parents and reviewed by pediatric experts. *ATEC* and a questionnaire were used for follow-up. The questionnaire asked about GI, diet, sleep, mood, and behavior. GI was scored by parents. The criteria were as follows: 0 points for no GI abnormalities, 1 point for occasional abnormalities (1–2 episodes of diarrhea or only 1 bowel movement over 2 days per week), 2 points for frequent abnormalities (3–5 episodes of diarrhea or 1 bowel movement over 2 days per week), and 3 points for long-term or severe abnormalities (more than 5 episodes of diarrhea or 1 bowel movement over 5 days per week).

### Statistical Analysis

Wilcoxon signed-rank test, paired wilcoxon test, and Kruskal-Wallis test analysis were performed using R [http://cran.r-project.org/]. All *p*-values reported in the study were from two-tailed tests and *p*-values lower than 0.05 were accepted as significant in clinical data analysis. All *p*-values for bacterial microbiome analyses were corrected using the Benjamini-Hochberg false discovery rate (FDR) correction, and the resulting corrected values were referred to as *q*-values. *q*-values lower than 0.05 were accepted as significant.

## Results

We here investigated 114 children with ASD and 40 NT children from 4 clinical centers. The average age of the participants was 4 years old (range: 3–8). The ratio of male to female was 1:1 in the NT group and 5:1 in the ASD group. There were no GI abnormalities in the NT group. In the ASD group, 61.4% (70/114) of the patients had GI events, with the most common being constipation, affecting 80% of participants in that group (56/70) (Table 1).

**Table 1:** Characteristics of study participants.

	**ASD**	**NT**
Subjects	114t	40
Age (mean)	4.5	4.2
**Gender**		
Female	19	20
Male	95	20
GI	70	0
Constipation	56	0
Diarrhea	7	0
other	7	0
Non-GI	44	33

*Data expressed as means with ranges when applicable. ASD, autism spectrum disorders subjects; NT, neurotypical subjects; NA, not applicable; ATEC, Autism treatment evaluation checklist*.

**Figure 1:**
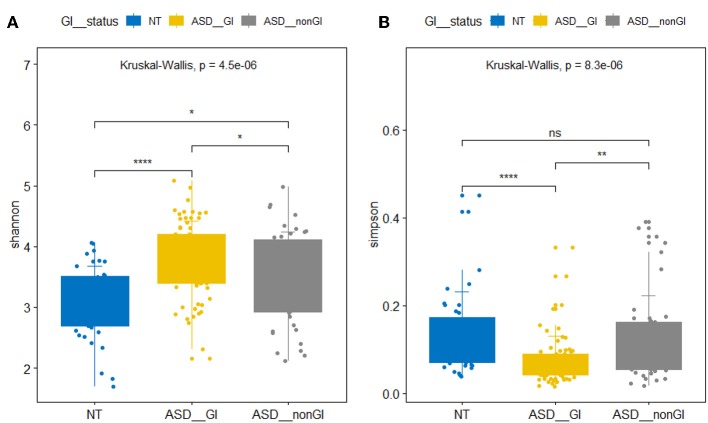
Box plots of α diversity of intestinal flora. **(A)** Shannon index. **(B)** Simpson index. NT represents neurotypical subjects. ASD_GI and ASD_nonGI represent autistic subjects with and without gastrointestinal symptoms, respectively (Kruskal-Wallis test, *p* < 0.05). ^*^P < 0.05, ^**^P < 0.01, ^****^P < 0.001.

We found significant differences between the ASD group and the NT group in the evenness of the intestinal microbiota and the relative abundance of the bacterial phyla and genus. The beta microbiota diversity index analysis did not yield significant results. In the α diversity analysis, the Shannon index of the ASD group with and without GI events was significantly higher than that of the NT group. However, the Simpson index of the ASD group with GI events was significantly lower than that of the NT group and the ASD without GI events, and no significant difference was observed between the NT group and the ASD without GI. The Simpson index is sensitive to microbiota evenness while the Shannon index is sensitive to the abundance of bacteria. Our results indicate that the ASD children in China had a higher intestinal microbiota abundance but a lower evenness (Figure 1). The ASD group without GI events showed a greater microbiota abundance than in the NT group and evenness similar to the NT group. At the phylum level, the relative abundance of *Bacteroidetes* and *Actinobacteria* in the ASD group was significantly lower than in the NT group, while the relative abundance of *Proteobacteria* in ASD group was higher than in the NT group. The *Firmicutes* in the ASD group was significantly greater than in the NT group. This result is consistent with results from previous research (9). At the genus level, the relative abundance of *Bacteroides, Bifidobacterium, Ruminococcus, Roseburia*, and *Blautia* in the ASD group was significantly lower than that in the NT group. However, *Lachnospira* was significantly more abundant than in the NT group (Table 2).

**Table 2:** Bacterial abundance at the level of phylum and genus in ASD and NT groups (Wilcoxon rank test *p*-value and *q*-value < 0.05).

	**ASD (mean)**	**NT (mean)**	***p*-value**	***q*-value**
**PHYLUM LEVEL**				
p_Bacteroidetes	0.12911	0.24565	7.15E-06	2.86E-05
p_Firmicutes	0.39346	0.59019	0.028254	0.064581
p_Actinobacteria	0.04222	0.07014	5.62E-05	0.00018
p_Proteobacteria	0.02626	0.04504	6.84E-12	1.09E-10
**GENUS LEVEL**				
g_Bacteroides	0.07772	0.18368	2.21E-07	2.13E-05
g_Bifidobacterium	0.03946	0.06787	3.66E-05	0.000813
g_Ruminococcus	0.01865	0.04330	7.77E-05	0.001536
g_Lachnospira	0.01844	0.05337	2.39E-07	2.13E-05
g_Roseburia	0.02674	0.05834	1.18E-06	5.25E-05
g_Blautia	0.01084	0.02157	0.000492	0.006256

We conducted a 4-week ABA training program in combination with probiotics treatment for 37 children with ASD, among whom 22 had GI events and 15 did not. Our results indicate that probiotics treatment, when applied in combination with ABA training, can alleviate the symptoms in both ASD children with and without GI illness. In 83.8% of ASD children, the *ATEC* total scores decreased by 8.1 points in average. Scores for Speech/Language Communication, Sociability, Sensory/Cognitive Awareness and Health/Physical/Behavior also significantly decreased. Among them, Health/Physical/Behavior scores declined the most. 86.7% (13/15) of ASD children without GI events showed improvement in *ATEC* scores. Their average *ATEC* score dropped from 59.3 to 50.2. The rate of improvement in *ATEC* score for children with ASD and GI abnormalities was 78.9% (17/22), and their average decrease in *ATEC* score was from 73.2 to 62.9. The rate of improvement in the ASD group without GI events was higher than that in the ASD group with GI abnormalities. *ATEC* scores showed no significant change in the control group (Table 3). Our results indicate that autistic children without GI symptoms are sensitive to probiotics treatment.

**Table 3:** Changes in *ATEC* and GI scores before and after 4-week probiotics treatment.

**Group (N)**	**Probiotics (37)**	**Probiotics NGI (15)**	**Probiotics GI (22)**	**Control (28)**
*ATEC* decrease (%)	83.8%	86.7%	78.9%	53.6%
*ATEC* total (mean)	67.1-59.0 (7.2 x 10^−7^)	59.3–50.2 (0.0013)	73.2-62.9 (1.7 x 10^−4^)	59.8-56.8 (0.89)
*ATEC* Speech/Language Communication (mean)	14.6-13.2 (1.9 x 10^−5^)	13.4-12.2 (0.014)	15.9–14.4 (3.6 x 10^−4^)	11.5-11.1 (0.93)
*ATEC* Sociability (mean)	16.6-14.3 (6.4 x 10^−5^)	14.9–12 (0.0057)	15.9–15.1 (0.0046)	15.8-14.2 (0.11)
*ATEC* Sensory/Cognitive Awareness (mean)	18.1-16.4 (0.0029)	15.8–14.1 (0.023)	20.0–17.4 (0.023)	16.1-15.9 (1)
*ATEC* Health/Physical/Behavior (mean)	17.8-14.2 (1.2 x 10^−4^)	15.2–11.8 (0.031)	19.7–15.9 (0.0016)	16.4–16.3 (0.83)
GI score decrease (%)	–	–	86.4%	–
GI score decrease (mean)	–	–	2.26–0.84 (1.6 x 10^−4^)	–

*N represents the total number of subjects in each group. The left and right sides of the “-” are the scores before and after probiotics treatment. The numbers in parentheses are p-values. GI and NGI indicate the presence and absence of GI symptoms, respectively*.

A gastrointestinal questionnaire was used to evaluate the ASD group with GI symptoms. In the treatment group, 19 out of 22 (86.4%) children have their GI score decreased. The mean GI score in the 19 cases decreased from 2.26 to 0.84. Scores in 3 cases remained unchanged. No significant improvements in GI score were found in the 11 children in the control group (Table 3).

We collected the observational questionnaire about children with ASD before and after probiotics treatment from parents. Among the 31 children who showed decreased *ATEC* scores, 17 also showed improvement in their behavioral symptoms (eye contact, obedience, self-injury behavior, etc.), 18 showed mood improvement (crying frequency, compliance), 19 patients showed improvement in eating-related symptoms (Improvement in appetite and pickiness), and 13 patients showed improvements in sleep quality. The behavioral and emotional improvement of ASD children with GI events was more pronounced than in children without GI events. ASD children without GI events showed more pronounced improvement in sleep quality and eating habits (Table 3).

During probiotics treatment, 2 patients developed diarrhea (2/37) 1 week after taking probiotics; the adverse reactions disappeared after the third week of continuous use. This may be because of the Jarisch-Herxheimer die-off reaction. No serious adverse reactions such as infection or exacerbation of symptoms occurred during the study. This demonstrates that a dosage of 30–40 billion CFU per day is suitable in this study.

## Discussion

Previous studies did not include participants of non-Western ethnicities and concentrated on ASD children with GI events. In this study, we recruited ASD children with and without GI symptoms from multiple centers in China. In this way, our research has a wide sample and better represents children with ASD. The ratio of male to female in our recruits was 1:1 in the NT group and 5:1 in the ASD group. The reason we think is that the incidence of ASD in boys is higher than girls, Early studies showed that autism affects 4–5 times more males than females (25). Our results indicate that there is a significant difference in the intestinal microbiota between ASD children and NT children regardless of their gastrointestinal problems. The abundance of beneficial bacteria in children with ASD was here found to be significantly lower than in non-ASD children. The abundance of *Bacteroidetes* in the ASD group was significantly lower than in the NT group, while the ratio of Firmicutes abundance to *Bacteroidetes* abundance was significantly higher than in the NT group. This suggests that although some ASD children do not exhibit gastrointestinal problems, their intestinal microbiota are, nonetheless, significantly different from those of non-ASD individuals.

Several studies and clinical trials have been conducted on the treatment of ASD with probiotics in attempt to reconstitute intestinal microbiota (26–28). However, they have been limited to participants of Western ethnicity. Some studies, despite better design than the above studies, have not drawn definite conclusions (29). Our study is the first report of probiotics treatment for Chinese ASD children. We have shown that after 4 weeks of treatment, more than 80% of ASD children's *ATEC* scores decreased. To our surprise, ASD children both with and without GI symptoms were found to be sensitive to probiotics treatment. In addition, a greater proportion of ASD children without GI symptoms responded to probiotics treatment than ASD children with GI symptoms. This is probably because ASD children without GI symptoms have less abnormal intestinal microbiota than ASD children with GI and are therefore more sensitive to probiotics treatment. Previous reports have shown that GI symptoms are correlated with the severity of ASD (15) ASD with GI may require longer treatment and higher doses of probiotics. Our results also show a significant decrease in the *ATEC* score in the probiotics group relative to the control group, which indicates that the probiotics can increase the intervention effect of ABA training on the child.

The strengths of our study are obvious. First of all, it is a multi-center clinical study of Chinese children. Secondly, the specimens we collected were children's feces, and there were no invasive and injurious operations on children. Thirdly, our findings include the results of probiotic intervention in some children. However, our study also has a few limitations. Our sample size is small, the duration of probiotics treatment was relatively short, and the study was not blinded. Future studies should be performed with larger sample sizes, longer treatment time, longer follow-up time, and more assessment parameters. Our research can be blinded by giving the ABA only group a pill placebo instead of a probiotic.

## Conclusions

Intestinal microbiota is different between the normal children and the ASD children with or without GI. Probiotics treatment can reduce the magnitude ASD-related and gastrointestinal symptoms. In combination with ABA training, probiotics treatment can bring benefits to ASD children with and without GI. Clinical trials with a more rigorous design and larger sample size are needed for validation.

## Data Availability Statement

Publicly available datasets were analyzed in this study. This data can be found here: http://www.chictr.org.cn.

## Ethics Statement

The studies involving human participants were reviewed and approved by Ethics Committee for Biomedical Research, First Hospital of Peking University. Written informed consent to participate in this study was provided by the participants' legal guardian/next of kin.

## Author Contributions

YH: full access to all the data in the study and takes responsibility for the integrity of the data and the accuracy of the data analysis. MN, YH, SZ, and QL: concept and design. All authors: acquisition, analysis, or interpretation of data and critical revision of the manuscript for important intellectual content. MN and YH: drafting of the manuscript. MN and SZ: statistical analysis. YH: obtained funding. JZ, FW, WD, GD, HL, WR, PY, CG, HT, XG, and SZ: administrative, technical, or material support.

### Conflict of Interest

The authors declare that the research was conducted in the absence of any commercial or financial relationships that could be construed as a potential conflict of interest.
